# Editorial 

**Published:** 2022-12

**Authors:** Ali Davoudi-Kiakalayeh

## Abstract

Floods in Asia, Europe, and America can lead to serious mortality and morbidity accounting for about 40% of all natural hazards. As a commonly occurring natural disaster in Iran, flood poses various risks and consequences. When this natural calamity occurs, the surface currents of water can cause drowning and trauma. It usually takes a long time for the flood to recede. People who experience drowning do not reach near-drowning because the golden time is lost until the water recedes so they have little chance of survival. Moreover, sharp objects floating in the flood water such as tree trunks or branches can be dangerous causing serious traumas. Rodents and reptiles like snakes may exacerbate the situation by entering residential areas and creating critical health problems. Water contamination is also an important issue at the time of floods because Escherichia coli, Salmonella, and Shigella contaminate the water sources and cause a great burden on the health system. Under such circumstances, people often suffer from hypothermia. Furthermore, the victims whose homes and assets have been damaged are at greater risk of mental illness. Thus, both the physiological and mental health of the people in flood-hit areas need special, careful consideration. But which one increases the risk of drowning? Hot or cold water? It is more likely to survive in cold and fresh water, and resuscitation measures can be performed better in cold water, because low temperature diminishes the metabolism, and slows down the death of tissues. In hot water, brain tissue is damaged in 5 minutes while in cold water it takes more than 10 minutes. The density of water in the lung is higher than that of the tissue and pulmonary ventilation becomes difficult.

The Fifth National Conference on Drowning Prevention was held with a focus on health in disasters with great support from the Guilan Road Trauma Research Center, Guilan University of Medical Sciences, Rasht, Iran. Forty universities, and faculties of medical sciences, as well as Deputy offices of the Ministry of Health and Medical Education, made important contributions to holding this national event. All announcements and registrations were performed via the Tasadofnews website (www.tasadofnews.ir). More than 1600 experts in the field, 260 doctors, and 400 nurses participated in this online event simulcasted on Apart and Ircme websites.

